# Dissection of drought response of modern and underutilized wheat varieties according to Passioura's yield-water framework

**DOI:** 10.3389/fpls.2015.00570

**Published:** 2015-07-23

**Authors:** Alireza Nakhforoosh, Heinrich Grausgruber, Hans-Peter Kaul, Gernot Bodner

**Affiliations:** Department of Crop Sciences, University of Natural Resources and Life SciencesVienna, Austria

**Keywords:** drought resistance, water use efficiency, genetic resources, phenology, stomatal conductance

## Abstract

Trait-based breeding is essential to improve wheat yield, particularly when stress adaptation is targeted. A set of modern and underutilized wheat genotypes was examined in a 2-year field experiment with distinct seasonal water supply. Yield formation and drought response strategies were analyzed in relation to components of Passioura's yield-water framework based on phenological, morphological, physiological, and root characteristics. Limited water supply resulted in 60% yield loss and substantially lower water use (37%), water use efficiency (32.6%), and harvest index (14%). Phenology and root length density were key determinants of water use. Late flowering underutilized wheat species with large root system and swift ground coverage showed greatest water use. Leaf chlorophyll concentration and stomata conductance were higher in modern cultivars, supporting their high biomass growth and superior water use efficiency. While, lower chlorophyll concentration and stomata conductance of underutilized wheats indicated a water saving strategy with an intrinsic limitation of potential growth. Harvest index was strongly dependent on phenology and yield components. Optimized flowering time, reduced tillering, and strong grain sink of modern cultivars explained higher harvest index compared to underutilized wheats. Cluster analysis revealed the consistent differentiation of underutilized and modern wheats based on traits underlying Passioura's yield-water framework. We identified physiological and root traits within modern cultivars to be targeted for trait-based crop improvement under water-limited conditions. High capacity of water use in underutilized genetic resources is related to yield-limiting phenological and morphological traits, constraining their potential role for better drought resistance. Still some genetic resources provide adaptive features for stress resistance compatible with high yield as revealed by high harvest index under drought of Khorasan wheat.

## Introduction

Grain yield is the product of numerous developmental processes during crop growth. It is a trait governed by multiple genes and highly influenced by environmental conditions. Yield improvement in water-limited environments is complex and depends strongly on the drought regime, i.e., drought duration, intensity, and time of occurrence (van Ginkel et al., 1998; Blum, 2011). This complexity becomes evident when attributes contributing to yield loss mitigation in a given environment are not equally useful in other water-limited environments (Richards, 2006). Despite these difficulties, wheat yield was remarkably increased over the second half of the 20th century in all wheat growing environments (Calderini and Slafer, 1998; Fischer et al., 2014) as a result of genetic improvement, enhanced input of production factors, particularly water and nitrogen (Sinclair and Rufty, 2012), and a synergy between them (Richards et al., 2014). However, in the last decades, rates of yield improvement in wheat have declined to less than what would be required to meet projected demands for 2050 (Hall and Richards, 2013). Particularly in dry regions, yield increase was below breeding progress registered for high yielding environments (Trethowan et al., 2002; Graybosch and Peterson, 2010).

Hitherto, only limited yield gains were realized using physiological traits for selection in drought prone environments (Richards, 2006; Reynolds et al., 2009). This is probably due to incomplete understanding of the physiological and genetic basis of drought resistance (Salekdeh et al., 2009) as well as insufficient consideration of drought environments when defining target traits for stress resistance (Rebetzke et al., 2013a). Also the upscaling of relevant drought defense mechanisms from the cellular level (e.g., dehydrins, Hassan et al., 2015; aquaporins, Maurel and Chrispeels, 2001) to the whole plant and stand level is challenging when searching for key traits in crop improvement.

The conceptual framework of Passioura (1977, 2006) facilitates the dissection of drought-adaptive mechanisms for trait-based breeding under drought-prone environments (Richards, 2006; Salekdeh et al., 2009). The framework relates yield under water limited conditions to (i) crop water use (WU), (ii) water use efficiency (WUE), and (iii) harvest index (HI). In the past, wheat grain yield improvement has largely been driven by improvements in HI rather than biomass (BM). Thus, HI is already close to its theoretical limit (Perry and D'Antuono, 1989; Shearman et al., 2005; Sadras and Lawson, 2011).

An option for yield improvement under drought stress is maximizing transpiration, i.e., better WU (Blum, 2009). This necessitates genotypes showing drought avoidance via uptake optimization, termed “water spenders” by Levitt (1980). In that respect, enhanced plant root systems are considered to be a promising approach (Wasson et al., 2012).

WUE as target trait was critically discussed by Blum (2009) because (i) WUE defined as BM/WU is not independent of WU, and (ii) it might go along with reduced crop transpiration and hence yield under moderate stress conditions. Passioura (2006), however, pointed to the single leaf scale of gas exchange as a key for WUE. Thereby, WUE can be considered (scale) independent from the whole plant WU within the original yield-water framework. High WUE of crops can be conferred by both stomata conductance and photosynthetic capacity. Udayakumar et al. (1998) suggested that only in those cases where high WUE is achieved via photosynthetic capacity, consistent yield increase could be expected. Condon et al. (2002) revealed that low stomata conductance as a reason for superior WUE generally expresses a conservative WU and leads to lower yields except for very dry environments where crop growth strongly relies on stored soil moisture.

Underutilized wheat species are valuable genetic resources for secondary drought-adaptive traits (Reynolds et al., 2007; Trethowan and Mujeeb-Kazi, 2008). Nakhforoosh et al. (2014) revealed significant genotypic diversity for root traits as well as for root functionality in terms of soil water depletion. Khazaei et al. (2010) demonstrated an essential influence of ploidy level on stomata size in Iranian wheat landraces.

Here we provide a comprehensive comparative analysis of modern and underutilized wheat germplasm based on their phenology, morphology, physiology, and root characteristics. The main objective is a trait based dissection of drought stress response strategies. We apply Passioura's yield-water framework and relate our investigated traits to the components of this analytical approach. Based on the identification of distinct strategies to cope with limited water supply, we will highlight strengths and weaknesses of underutilized wheat germplasm for trait-based breeding under water-limited conditions.

## Materials and methods

### Plant material

Wheat genotypes of different ploidy levels, origins, and breeding intensities were examined in a 2-year field experiment. In 2011 seven durum wheat (*Triticum turgidum* subsp. *durum* (Desf.) Husnot), two Khorasan wheat (*T. turgidum* subsp. *turanicum* (Jakubz.) Á. Löve and D. Löve), two einkorn wheat (*T. monococcum* L. subsp. *monococcum*), and one Zanduri wheat (*T. timopheevii* (Zhuk.) Zhuk. subsp. *timopheevii*) were tested. In 2012, six contrasting genotypes from the previous year were examined along with two common wheat (*T. aestivum* L. subsp. *aestivum*) and one Persian wheat (*T. turgidum* subsp. *carthlicum* (Nevski in Kom.) Á. Löve and D. Löve) (Table 1). The six genotypes which were tested in both years are hereinafter referred to as “core set.”

**Table 1:** **Characteristics and origin of the wheat germplasm used in the experiment**.

**Year/Genotype**	**Origin^a^**	**Donor/Breeder**	**Ploidy/Genome**	**Species**
			****	
**2011**				
SZD3146	AT	Saatzucht Donau, AT	4×, BA^u^	durum
Clovis	FR	GIE Eurodur, FR	4×, BA^u^	durum
7060; 7063; 7094^b^	MX	CIMMYT, MX	4×, BA^u^	durum
TRI5254	?	IPK Gatersleben, DE	4×, BA^u^	Khorasan
**2011–2012**				
QK-77 (Kamut®)	US	AGES, Vienna, AT	4×, BA^u^	Khorasan
Floradur	AT	Saatzucht Donau, AT	4×, BA^u^	durum
Matt	US	Arizona Plant Breeders, US	4×, BA^u^	durum
PI428154; PI428165	TR	NSGC, Aberdeen, US	2×, A^m^	einkorn
W9	GE	GSAU, Tbilisi, GE	4×, GA^m^	Zanduri
**2012**				
W13	GE	GSAU, Tbilisi, GE	4×, BA^u^	Persian
Tabasi	IR	IFA Tulln, AT	6×, BA^u^D	common
Taifun	DE	KWS Lochow GmbH, DE	6×, BA^u^D	common

*^a^ AT, Austria; DE, Germany; FR, France; GE, Georgia; IR, Iran; MX, Mexico; TR, Turkey; US, United States*.

*^b^ Entry codes of the 40th IDSN (International durum wheat screening nursery)*.

### Experimental conditions

Field experiments were carried out under rainfed conditions in Raasdorf (48°14′N, 16°35′E, 156 m) in the Pannonian plains of Austria. Long-term (1981–2010) annual precipitation and mean temperature are 516 mm and 10.3°C, respectively. Daily weather data were obtained from a weather station located at the trial site. According to WRB (IUSS, 2014) soil is a chernozem with silt loam texture (sand 0.21 kg kg^−1^; silt 0.57 kg kg^−1^; clay 0.22 kg kg^−1^) with high water holding capacity (water content at field capacity: 0.286 cm^3^ cm^−3^, water content at wilting point: 0.118 cm^3^ cm^−3^).

Hydrological site conditions were characterized using the HYDRUS 1D software package (Šimůnek et al., 2013). The objectives of model based environmental characterization were (i) to define moisture conditions during the study years in relation to longtime site hydrology, and (ii) to provide a hydrological basis for the analysis of trait based stress response.

The field experiments were sown on 8th March 2011 and 20th March 2012 in a four replicate randomized complete block design following a shallow seedbed preparation using a rotary harrow. Sowing was carried out by a plot seeder (Wintersteiger, Ried, Austria) with a seeding rate of 400 seeds per m^2^. Plot size was 7.5 m^2^ with 10 rows spaced 12.5 cm apart. The site has high availability of P and K and was fertilized with 100 kg ha^−1^ N to exclude nutrient limitation.

### Phenotypic measurements

#### Yield and yield components

After full ripening (BBCH 92), plants were hand harvested from a 0.25 m^2^ area from the center of each plot. Total aboveground biomass, seed yield (oven dried at 60°C for 48 h), number of fertile tillers, and number of seeds per ear were measured and expressed per unit area. Thousand grain weight was determined by weighing 400 seeds.

Sensitivity of genotypes to water limitation was characterized by relative stress response (RSR) of traits between the two experimental years which differed strongly in seasonal water supply. RSR of yield and its components was calculated as:

(1) $$RSR\;=\;\frac{Trait_{wet}\;-\;Trait_{dry}}{Trait_{wet}}$$

where *Trait*_*wet*_ is the trait value under high water availability (i.e., 2011) and *Trait*_*dry*_ is the value under low water availability conditions (i.e., 2012).

#### Water use traits

Water use was calculated as a simplified water balance from soil water depletion (ΔS̄) and cumulative rainfall. ΔS̄ was defined as the difference in soil water storage between sowing and harvest. Soil water content (θ) was measured weekly every 10 cm down to 90 cm soil depth by a capacitance probe (Diviner 2000®, Sentek Pty Ltd., Stepney, Australia). Surface runoff can be neglected at the present experimental site. Deep drainage can't be quantified from soil water content measurements. From lysimeter studies at the site, however, it is known that due to low amount of rainfall and high soil water holding capacity, the amount of seepage water during the growing season is negligible (Nolz et al., 2014). The term WU_ET_ is used to indicate that water use includes both plant transpiration as well as soil evaporation. In a water balance approach these two components can't be measured separately.

Phenology was assessed using the BBCH decimal code (Lancashire et al., 1991). Time to any developmental stage was expressed in cumulative thermal time (CTT), measured in degree-days (°C d) as described by Salazar-Gutierrez et al. (2013) and assuming a constant base temperature (*T*_*b*_) of 0°C as no information was available on possible *T*_*b*_ differences among genotypes.

Ground cover by leaf area was measured by digital imaging twice at early emergence and when canopy almost closed using a Canon EOS20D (Canon Inc., Tokyo) digital camera at 1.5 m height above the canopy. Digital images were analyzed individually by SigmaScan Pro Vers. 5.0 software (SystatSoftware Inc., Chicago) to identify green leaves and calculate the percentage of green ground cover as described by Richardson et al. (2001). Ground cover rate as an indicator of early vigor was calculated as the difference in ground cover between the two measurements divided by the CTT of the corresponding period. Ground cover rate could be hypothesized as a water use driver because of (i) higher early demand due to quicker leaf area development, (ii) possibly a related higher early rooting vigor, and (iii) higher allocation of available water to plant transpiration than soil evaporation.

Root morphological traits were measured from soil cores and subsequent image analysis. In this study we will only refer to selected root data (i.e., root length density and root-to-shoot ratio). Details on root sampling and root system characterization are given in Nakhforoosh et al. (2014).

#### Water use efficiency traits

Following Blum (2009), water use efficiency was dissected into biomass and water use, i.e., water use efficiency for biomass (WUE_b_) equals BM/WU_ET_. Investigated traits related to WUE_b_ were photosynthetic capacity and stomata conductance. Photosynthetic capacity was approximated by leaf chlorophyll content measured at heading (BBCH 50) using a SPAD-502 Plus chlorophyll meter (Konica Minolta Holdings, Inc., Tokyo). Ten plants were randomly selected in each plot and SPAD values of the flag and/or penultimate leaf of the main stem were recorded at 5 points along the proximal-distal axis of the leaf. Stomatal conductance was measured using an AP4 porometer (Delta-T Devices Ltd., Burwell, Cambridge, UK) in parallel with SPAD measurements at BBCH 50.

### Statistical analysis

Analyses of variance for single years and combined analyses across years were performed using the MIXED procedure of SAS 9.2 software (SAS Institute, Inc., Cary, NC). Genotypes were treated as fixed effects, block, block (year), year, and/or genotype by year interaction as random effects. The best linear mixed models were selected according to the corrected Akaike information criterion (AICC).

To study direct and indirect relations of observed traits with the components of Passioura's framework regression analysis was applied via the REG procedure of SAS. Procedure CLUSTER was applied to determine similar groups of genotypes based on yield components, components of Passioura's framework and the underlying phenological, morphological, and physiological traits (Bodner et al., 2013).

## Results

### Rainfall pattern and soil water availability

Hydrological conditions at the experimental site are displayed in Figure S1 (Supplementary Material). Longtime rainfall during the vegetation period of spring cereals is 237 mm, while plant available water (PAW) in the soil from stored winter moisture at time of sowing is 170 mm, i.e., 42% of total seasonal crop water supply (Figure S1A). Monthly in-season rainfall increases toward summer, resulting in a favorable balance between climatic demand and supply. Therefore, the site can be described as predominantly supply driven.

**Figure 1:**
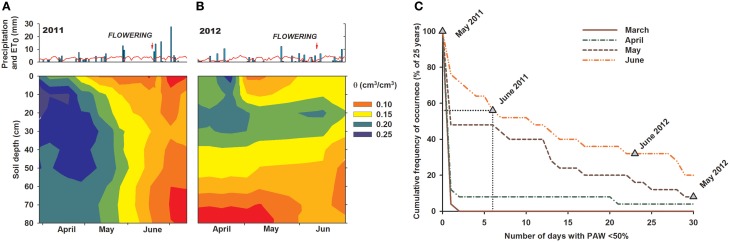
**(A,B)** Daily rainfall and potential evapotranspiration (ET0) at the experimental site over 2011–2012 (Modified after Nakhforoosh et al., 2014), and **(C)** cumulative frequency of occurrence of days with < 50% plant available water (PAW) over 1m soil profile depth during growing season. Calculation based on annual vs. longtime soil water content simulation. Triangles indicate the number of days observed in 2011 and 2012 with < 50% PAW. Dotted line exemplifies interpretation for June 2011: 6 stress days with < 50% PAW means that 56% of years have ≥6 stress days, while 44% of years have < 6 stress days, that means June 2011 is within the wetter half of years for this site.

The two experimental years showed distinct hydrological conditions (Figures 1A,B). Although annual mean temperature and precipitation were similar (2011: 10.5°C, 395 mm; 2012: 10.9°C, 402 mm), in-season rainfall distribution and stored soil moisture at sowing differed strongly. During May and June, i.e., time of stem elongation, heading, anthesis, and early grain filling, rainfall was significantly lower in 2012 (77 mm) than in 2011 (113 mm). Differences in previous autumn precipitations (2010: 308 mm; 2011: 49 mm) resulted in substantially lower simulated PAW on 1st March in 2012 compared to 2011.

Based on the simulated long-term site hydrology and the measured soil moisture pattern during the experiment, we determined the number of stress days using a threshold of ≤50% PAW and calculated the probability of occurrence of the two experimental years compared to the last 25 year average (1988–2013) (Figure 1C). Seasonal water availability revealed that in 2011 hydrological conditions in May were among the wetter half of years, while June water availability was similar to 65% of years. Contrary, 2012 was a particularly dry year with a low probability of occurrence in 25 years. Due to low water storage over winter and reduced rainfall in spring, prolonged dry periods with water contents below 50% PAW were observed in May and June. The probability of occurrence of dry conditions of similar intensity as in 2012 is 8% for May and 32% for June, respectively.

Thus, site hydrology revealed that only limited water stress occurred in June 2011, whereas 2012 was a particularly dry year with high stress incidence. Consequently, changes in crop performance between the 2 years can be interpreted in terms of drought response.

### Yield and yield components

Significant (*P* < 0.001) genotypic variation was observed for grain yield and all other yield components. Combined ANOVA of the core set revealed also significant variation for year and genotype × year interaction (Supplementary Table S1).

Grain yield varied from 209.2 (TRI5254) to 541.3 g m^−2^ (7060) and 37.9 (PI428154) to 237.7 g m^−2^ (“Floradur”) in 2011 and 2012, respectively (Table 2). Mean drought-induced grain yield loss in 2012 was 60.6% for the core set. Adapted durum “Floradur” showed the highest grain yield among core set genotypes followed by early flowering “Matt” and Khorasan wheat “QK-77” (Kamut®), whereas *T. monococcum* and *T. timopheevi* accessions were lowest yielding. Yield reduction in 2012 was lowest for Khorasan (20.5%), intermediate for “Matt” and “Floradur” (51.2 and 54.7%, respectively) and highest for the einkorn and Zanduri wheat (80.1–85.2%).

**Table 2:** **Genotypic mean values of grain yield and yield components in 2011 and 2012**.

**Year/Genotype**	**YLD^a^ (g m^−2^)**	**BM (g m^−2^)**	**HI**	**TIL_f_ (n m^−2^)**	**SPE (n)**	**TGW (g)**	**PH (g)**
**2011**							
7060	541.3	1171.2	0.46	354.3	36.5	41.8	66.3
7063	472.5	1087.5	0.43	324.0	29.2	50.8	73.8
7094	404.1	939.4	0.43	310.1	29.8	44.8	68.8
Clovis	413.9	941.4	0.43	296.7	25.9	54.0	73.8
Floradur	525.0	1165.4	0.45	383.3	28.1	48.7	72.5
Matt	365.9	810.6	0.45	286.7	26.3	48.4	60.0
SZD3146	395.9	908.6	0.44	302.1	25.6	51.9	72.5
Kamut	254.2	856.3	0.29	227.6	17.1	67.2	105.0
TRI5254	209.2	615.1	0.34	248.6	16.8	52.5	107.5
W9	256.4	1001.9	0.26	530.8	17.5	27.8	70.0
PI428154	240.5	909.6	0.26	759.6	12.7	25.2	67.5
PI428165	247.5	926.0	0.27	994.8	11.4	21.8	71.3
s.e.d.^b^	55.8	111.7	0.016	56.2	2.8	2.5	2.2
d.f.	33	33	36	33	33	33	33
**2012**							
Tabasi	141.3	348.9	0.40	276.9	13.2	38.2	70.3
Taifun	126.4	387.6	0.33	325.0	11.2	35.3	61.0
Floradur	237.7	504.4	0.47	362.5	18.1	36.1	63.3
Matt	178.9	402.4	0.44	295.2	14.5	42.5	50.0
Kamut	202.1	481.7	0.42	261.6	15.7	49.4	79.8
W13	152.0	373.2	0.41	345.2	17.5	25.2	78.8
W9	37.9	368.2	0.11	355.8	4.2	25.7	74.8
PI428154	37.9	326.7	0.12	447.1	4.2	20.7	73.3
PI428165	49.3	328.8	0.15	527.9	4.7	20.1	69.0
s.e.d.	21.2	49.4	0.022	37.8	1.5	1.4	3.8
d.f.	27	27	27	27	27	24	24

*^a^ YLD, grain yield; BM, shoot biomass; HI, harvest index; TIL_f_, number of fertile tillers; SPE, seeds per ear; TGW, thousand grain weight; PH, Plant height*.

*^b^s.e.d., standard error of differences; d.f., degrees of freedom*.

Einkorn and Zanduri wheat showed a significantly higher number of fertile tillers, whereas Khorasan wheat had the lowest tillering capacity. Number of seeds per ear varied from 11.4 (PI428165) to 36.5 (7060) and 4.2 (PI428154) to 18.1 (“Floradur”) in 2011 and 2012, respectively. Elite durum germplasm had a significantly higher number of seeds per ear compared to underutilized wheats, showing that seed number is a key component for high yielding cultivars. *T. turanicum* and *T. monococcum* showed the largest and smallest seed weight, respectively. Particularly for Khorasan wheat, seed weight was the component ensuring a relatively high yield.

When analyzing the sensitivity of the core set genotypes for yield components in response to low water availability, it is evident that seed number per ear and tillering were highly sensitive, especially for the neglected species einkorn and Zanduri wheat with a RSR of 68.5 and 41.8%, respectively, while they had a relatively stable seed weight (11%). Durum varieties had high sensitivity for seeds per ear (40%) followed by seed weight (19.1%). Khorasan wheat responded to drought stress mainly with seed weight loss (26.5%) along with plant height reduction (Table 2).

### Components of passioura's yield-water framework and related traits

**Figure 2:**
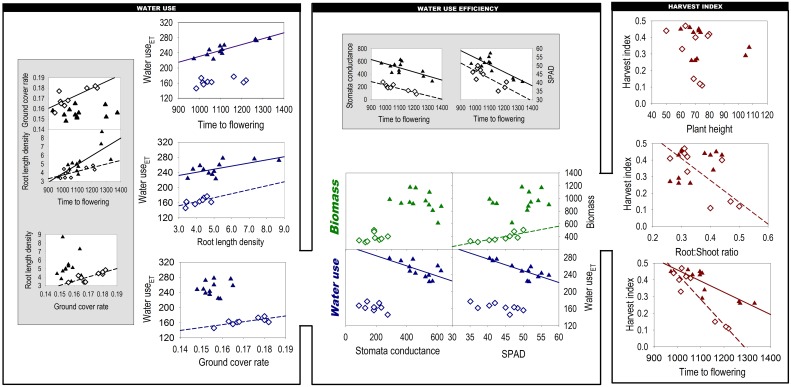
**Phenological, morphological, physiological, and root traits underlying the components of Passioura's yield-water framework**. Figures on gray background indicate secondary inter-trait associations. (Closed triangles: 2011; open diamonds: 2012; regression lines indicate significant relations). Units are: Ground cover rate % Cd^−1^, Root length density cm cm^−3^, Time to flowering °Cd, Water use_ET_ mm, Stomata conductance mmol m^−2^ s^−1^, Biomass g m^−2^, SPAD dimensionless, Harvest index g g^−1^, Plant height cm, Root:Shoot ratio g g^−1^.

Figure 2 shows the components of the yield-water framework according to Passioura (1977) and relations to traits that we hypothesized to constitute the crops' phenological, morphological and physiological drivers of WU, WUE, and HI. Beside direct relations of traits with Passioura's components, we also provide some secondary inter-trait relations suggesting hierarchical dependences among traits.

Water use varied significantly between genotypes in each year and ranged from 223.2 (7063) to 277.8 mm (W9) and from 145.9 (“Matt”) to 177 mm (PI428165) in 2011 and 2012, respectively (Table 3).

**Table 3:** **Genotypic mean values of phenological and physiological traits in 2011 and 2012**.

**Year/Genotype**	**WU^a^_ET_(mm)**	**ANTH1(°Cd)**	**ANTH2(d)**	**GCR(% ° Cd^−1^)**	**SPAD**	**SC(mmol m^−2^s^−1^)**	**WUE_b_(g m^−2^ mm^−1^)_b_**
**2011**							
7060	247.8	1045.3	85.5	0.148	48.2	420.8	4.78
7063	223.2	1065.0	86.5	0.159	50.4	547.8	4.92
7094	258.0	1096.6	88.0	0.165	50.0	418.2	3.76
Clovis	234.7	1035.6	85.0	0.154	52.2	522.9	4.17
Floradur	242.9	1096.6	88.0	0.154	51.0	457.5	4.86
Matt	224.3	972.3	81.5	0.158	48.0	569.3	3.67
SZD3146	238.2	1106.5	88.5	0.152	53.4	544.7	3.84
Kamut	248.6	1106.5	88.5	0.151	51.5	625.1	3.51
TRI5254	260.1	1116.5	89.0	0.156	49.2	603.7	2.39
W9	277.8	1331.4	100.0	0.156	41.2	287.0	3.63
PI428154	272.0	1269.4	97.0	0.152	38.7	362.4	3.39
PI428165	275.7	1264.6	96.8	0.164	44.9	440.9	3.41
s.e.d.^b^	20.7	7.9	0.4	0.004	1.8	60.1	0.65
d.f.	33	36	36	36	36	36	33
**2012**							
Tabasi	173.3	1007.5	76.0	0.177	45.0	219.5	2.03
Taifun	161.4	1014.7	76.5	0.167	47.8	190.2	2.48
Floradur	156.3	1018.4	76.8	0.165	54.0	184.5	3.33
Matt	145.9	983.5	74.8	0.156	48.9	275.2	2.73
Kamut	163.5	1037.4	78.0	0.163	48.6	185.2	2.97
W13	162.6	1056.3	79.0	0.168	44.2	233.4	2.33
W9	167.2	1219.4	88.0	0.180	38.2	86.6	2.21
PI428154	161.7	1208.3	87.5	0.182	34.8	128.0	2.03
PI428165	177.0	1161.7	85.0	0.180	37.0	140.5	1.87
s.e.d.	12.6	13.2	0.7	0.005	1.9	29.3	0.40
d.f.	24	27	27	27	27	24	24

^a^ WU_ET_, water use; ANTH1, anthesis (BBCH 65) based on cumulative thermal time (CTT); ANTH2, anthesis based on calendar time (days after flowering); GCR, ground cover rate (between emergence and almost closed canopy); SPAD, leaf chlorophyll content (SPAD values); SC, stomatal conductance; WUE_b_, Water use efficiency for biomass;

*^b^ s.e.d., standard error of differences; d.f., degrees of freedom*.

**Figure 3:**
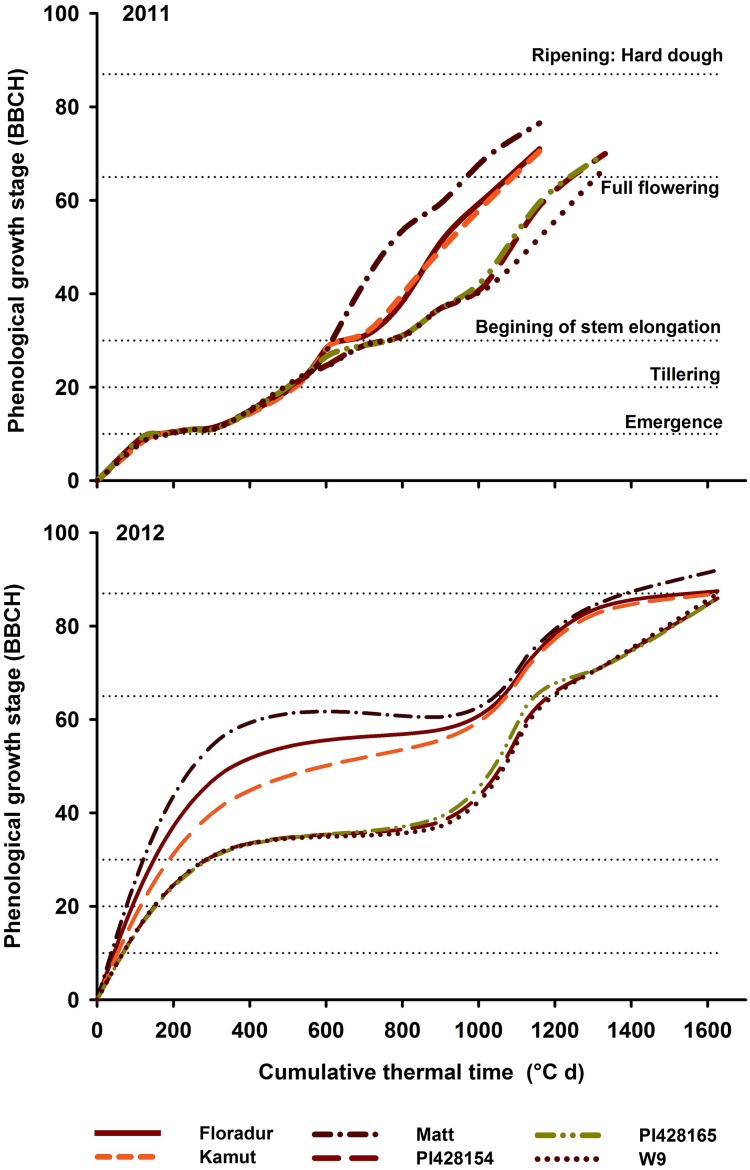
**Genotypic variation in phenological growth development of core set genotypes in 2011 and 2012**. In each year the cumulative thermal time (CTT) was calculated from the day of sowing onwards.

For all phenological and physiological traits (Table 3) a significant (*P* < 0.05) difference between genotypes was observed. Water shortage in 2012 resulted in a 37% reduction in average WU_ET_ among core set genotypes (i.e., from 256.9 in 2011 to 161.9 mm in 2012). Einkorn wheat PI428165 (226.3 mm) and durum cv. “Matt” (185.1 mm) showed the highest and lowest WU_ET_ over the 2 years. With respect to flowering the genotypes can be classified into three groups (Figure 3, Table 3): (i) early flowering durum cv. “Matt,” (ii) intermediate flowering group incl. “Floradur” and other tetraploid and hexaploid wheat genotypes, and (iii) very late flowering underutilized wheats *T. monococcum* and *T. timopheevi*.

In the core set, flowering hastened in 2012 (2011: 91.9 d; 1173.5°Cd; 2012: 81.7 d, 1104.8°Cd). This was more evident with respect to early growth stages, i.e., from emergence to stem elongation. Late flowering wheat relatives inevitably showed shorter grain filling periods than durum wheat over 2 years.

The relation of time to flowering with WU_ET_ was significant in 2011, when wetter soil profile alongside with in-season rainfalls provided appropriate conditions for longer root water uptake of late flowering genotypes. However, in 2012, low water availability in May and June obviously restricted prolonged water extraction by late flowering varieties and hence reduced variation in WU_ET_.

Early vigor, as determined by ground cover rate, was significantly higher for the core set in 2012 than 2011 (0.171 vs. 0.156% °C d^−1^, respectively) with a significant genotype by year interaction (Table S1). Despite an initial lag phase, which was especially evident for *T. timopheevi* W9, underutilized wheat relatives closed their canopy more swiftly than durum and Khorasan wheat, particularly in 2012.

In both years a strong relationship between root length density and WU_ET_ was observed. This indicates that roots are key determinant for the WU_ET_ component in Passioura's framework. Differences in root length density and other root parameters are presented in detail in Nakhforoosh et al. (2014).

Water use efficiency (WUE_b_) showed significant differences among the germplasm in both years. “Floradur” along with durum lines 7060 and 7063 had highest WUE_b_ in 2011. “Floradur” remained superior in WUE_b_ also in 2012. Genotypes with lowest WUE_b_ were Khorasan wheat TRI5254 in 2011 and einkorn wheat along with Iranian wheat “Tabasi” in 2012. Average WUE_b_ of the core set dropped from 3.7 to 2.5 g m^−2^ mm^−1^. “QK-77” was the most stable genotype of the core set in sustaining WUE_b_ (15.4%) while “Floradur” (31.5%) and “Matt” (25.6%) had an intermediate response. Underutilized wheats were most susceptible to drought stress (41.4%).

Stomatal conductance declined substantially for the core set in response to water scarcity in 2012 (i.e., from 457.0 to 166.6 mmol m^−2^ s^−1^). In 2011 Khorasan wheat (TRI5254, Kamut®) along with durum cv. “Matt” showed highest stomatal conductance while einkorn and Zanduri wheat were characterized by the lowest stomatal conductance. In 2012, “Matt” and *T. carthlicum* W13 had highest stomatal conductance whereas, like in 2011, the underutilized species *T. monococcum* and *T. timopheevii* showed the lowest stomatal conductance.

Chlorophyll concentration, as an indicator for photosynthetic capacity measured by SPAD, showed a significant decrease for the core set in 2012 which was more evident for underutilized wheat species. In 2011, durum wheats SZD3146 and “Clovis” were the genotypes with highest chlorophyll content followed by “QK-77” and “Floradur,” while in 2012 “Floradur” was the superior genotype. Accessions of einkorn and Zanduri wheat constantly had the lowest SPAD values in both years.

Both WUE_b_ components (biomass, water use) were influenced by the measured physiological leaf traits. Water use showed a negative association with both stomatal conductance and chlorophyll content, being significant in 2011 only. Water use of late-maturing einkorn and Zanduri wheat was higher, in spite of lower stomatal conductance. Biomass showed a significant relation with leaf chlorophyll content in the dry year 2012, suggesting this measurement as an appropriate indicator for WUE_b_ under limited water condition. Similar to stomatal conductance, leaf chlorophyll content was positively related to earliness in both years.

In 2011 the highest harvest index (HI) values were observed for durum wheat (mean 0.42), followed by Khorasan wheat (0.32) and the underutilized wheat species *T. monococcum* and *T. timopheevii* (0.26). In 2012 HI of the latter underutilized wheat species decreased significantly (0.13) in response to drought, while “Floradur” and “Matt” almost retained their HI. Interestingly, Khorasan wheat “QK-77” showed even an increase in HI. With respect to plant height *T. turanicum* was significantly taller than the other wheat species. Significant genotypic variation for root-to-shoot was observed only in the dry year 2012 (Nakhforoosh et al., 2014). HI was negatively associated with root-to-shoot ratio in this year.

### Trait based grouping of genotypes

**Figure 4:**
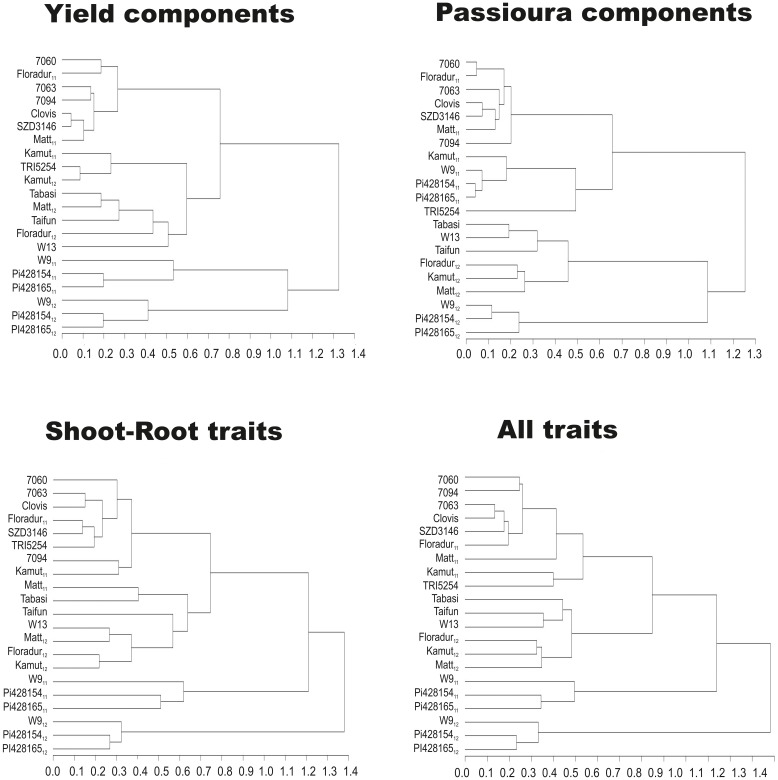
**Hierarchical clustering of wheat genotypes based on yield components, components of Passioura's yield-water framework, phenological, morphological, physiological, and root traits related to components of Passioura's yield-water framework, and all traits**.

Association between genotypes (and years) based on (i) yield components, (ii) Passioura components (WU_ET_, WUE_b_, HI), (iii) phenological, morphological, and physiological traits related to Passioura's components, and (iv) all traits was revealed by cluster analysis (Figure 4). Including all genotypes reveals the strength of group linkage driven by genotypic similarity (constitutive) and environmental influence (adaptive), respectively. Using different clustering variables shows which group of traits mainly expresses constitutive or adaptive linkage between genotypes.

The distinction between underutilized einkorn and Zanduri wheat and the other genotypes appeared at the highest hierarchy with the exception of Passioura components. In this case the first grouping was according to years, which is explained by the strong water dependence of these traits. At a lower hierarchical level four clusters can be distinguished, subdividing the whole sample according to years and/or wheat species. For clustering based on yield components a differentiation at a lower level is even obvious between durum and Khorasan wheat. With respect to Passioura components only four main clusters can be distinguished. Interestingly, Khorasan wheat “QK-77” changes the group between years: in 2011 (high water availability) “QK-77” is grouped together with the other underutilized wheat species *T. monococcum* and *T. timopheevii*, while in 2012 (low water availability) it joins the group of modern durum cultivars. The most meaningful grouping at high and low distances is provided when considering all traits. Here, in 2011 *T. durum* and *T. turanicum* are grouped in different clusters. Among the 2012 clusters, hexaploid wheats are next to each other, while einkorn and Zanduri wheat form distinct groups.

## Discussion

### Drought environment characterization

Understanding crop response to drought and relevant traits conferring better stress resistance requires a precise environmental characterization (Blum, 2011). Simulation models have been shown to be an appropriate tool for a proper description of the target environment for crop management and breeding activities (Chauhan et al., 2013). Continental climates as found in central-eastern Europe are distinguished by a higher proportion of in-season rainfall compared to stored soil moisture as source of crop water supply. Thereby, they differ essentially from storage driven Mediterranean winter rainfall climates or subtropical sites where dry season crops grow on residual soil moisture. Still stored water can be essential to buffer temporary dry periods affecting crop yield particularly when their occurrence coincides with sensitive growth stages.

The substantial change of crop performance due to low stored soil moisture together with low precipitation around flowering in our experiment clearly reveals that average climate variables (e.g., annual or seasonal rainfall sum) are insufficient to provide an appropriate picture on crop water stress.

According to Blum (2009) an efficient use of available soil water should be targeted as selection criteria. In this regard site hydrology determines which plant traits support most effective water uptake. Generally rooting depth is considered the key trait for superior plant water supply (e.g., Wasson et al., 2012; Lynch, 2013). However, Nakhforoosh et al. (2014) demonstrated that dense root systems in the upper soil layers rather than deep rooting provide highest plant water uptake in an environment with high in-season rainfall and high storage soils. This is particularly valid for dry years with a lack of subsoil moisture from off-season winter rainfalls, when investing into deep rooting is of limited value to sustain high transpiration. Also Tron et al. (2015) in a modeling study could show that in strongly supply driven environments, rooting density can become more important for plant water acquisition compared to rooting depth. These findings are in agreement with results from an ecological study by Sperry and Hacke (2002) in a desert environment with soils of different storage capacity revealing that exploitative root traits (e.g., rooting density, root xylem cavitation resistance) allowed better adaptation than exploitative traits (e.g., deep rooting) when soil water availability was higher in the top soil compared to deep soil layers. There appear two keys to a water efficient root system: (i) spatio-temporal synchronization of root distribution with the distribution of available soil water (Schenk and Jackson, 2002) and (ii) high root hydraulic functionality to efficiently exploit available water in accordance with crop needs (Vadez, 2014).

### Plasticity of yield components

Trait based strategies for better drought resistance in cereal crops require downscaling yield reduction under stress to the sensitivity of single yield components. In our study for example, “QK-77” (Kamut®) stabilized its grain yield at the cost of shoot biomass via a significant decrease in plant height, resulting in an increased HI, suggesting a potential for partitioning of biomass to seeds as an important stress adaptive trait frequently found for cereals (Blum, 1998; Shearman et al., 2005; Dreccer et al., 2009). Reduction of competition from alternative sinks (stem and infertile tillers) is hypothesized as an opportunity to increase the partitioning to spikes and further increase HI beyond its current limit (Foulkes et al., 2011). Although “QK-77” can be considered a water stress tolerant genotype, it does not show high yield potential under favorable water condition. Contrary, *T. monococcum* and *T. timopheevi* significantly reduced the number of fertile tillers, their main yield component, in response to suboptimum water availability. Number of seeds per ear and seed weight, which are both related to grain sink strength (Miralles and Slafer, 2007; Acreche and Slafer, 2009), are very low in these species, resulting in significantly lower HI despite reasonable biomass production. Restricting tillering capacity is considered beneficial where water limitation requires a more conservative uptake strategy over the growing season to provide the crop with enough water during grain filling (Richards et al., 2010). The main yield component of durum varieties was number of seeds per ear followed by seed weight, whereas number of fertile tillers showed no plasticity. Slafer et al. (2014) recommended a balanced dependence of grain yield on single components to ensure both high yield potential and sufficient plasticity in response to water limitation.

### Shoot and root traits underlying passioura's yield-water framework

Clustering genotypes based on Passioura's components revealed a clear distinction between tetraploid *T. turgidum* and underutilized *T. monococcum* and *T. timopheevi*, which was also demonstrated by their mean performances (Table S2). On the other hand, Khorasan wheat, a *turgidum* subspecies genetically similar to durum wheat but with lower breeding intensity, was more variable between and within clusters (Figure 4).

#### Water use traits

Phenology was a major distinction among genotypes and a key driver of other morphological and physiological traits. The prolonged vegetative development of *T. monococcum* and *T. timopheevi* is obviously genetically determined. But also breeding history and origin can result in significant different phenology, e.g., “Matt” vs. “Floradur” (Figure 3). Beside the constitutive differences among genotypes, there is also phenological plasticity in response to water availability. In 2012 the transition from vegetative into reproductive phase was obviously stimulated by water stress. Plasticity of early growth stages until stem elongation is well-known in wheat while later growth stages are generally more stable (e.g., McMaster and Wilhelm, 2003).

Flowering is the most sensitive stage to water shortage (Farooq et al., 2012). Progress has been achieved by breeding for earliness allowing crops to escape terminal drought stress and access enough soil water during flowering and grain filling (Salekdeh et al., 2009). However, vigorous growth and sufficient biomass prior to flowering is also critical for yield potential. In the present in-season rainfall environment yield limitation due to earliness was clearly demonstrated by low grain yields of early maturing cv. “Matt” compared to other advanced varieties and/or breeding lines. Grain yield of early maturing genotypes is largely limited by the potential number of grains per unit area which is determined between stem elongation and post-anthesis (Slafer et al., 2014). Among other factors, an overall lower water use seems to limit yield potential of very early cultivars, which can be attributed to a reduced rooting intensity (Mitchell et al., 1996).

The dominant morphological difference within the investigated germplasm was the number of fertile tillers. *T. monococcum* and *T. timopheevii* exhibited a high number, *T. turgidum* subsp. *durum* an intermediate number and *T. turgidum* subsp. *turanicum* a low number. In 2012 water stress resulted in a reduced number of tillers. Highest plasticity with respect to number of tillers was found for genotypes of the “high tillering” group. In regard to water use, tillering is relevant due to the secondary nodal root system developing from tillers (Zobel and Waisel, 2010). Thus, a shortened period between emergence and stem elongation with limited tillering can also limit the development of nodal roots, resulting in lower water use. The consistent association between water use and root length density confirms this relationship (Figure 2). On the other hand, the high tiller number of underutilized wheats is evidently limiting yield as revealed by its negative correlation to harvest index. Therefore, optimization of tiller, related to nodal rooting, for high water uptake is constraint within tight limits. Other root system traits such as increased fine rooting in response to drought might provide alternatives to improve water use (Nakhforoosh et al., 2014). Such an adaptive response is more compatible to high yields than alteration of assimilate allocation between roots and shoots.

An interesting trait promoting water use under conditions of limited availability was early vigor. Rapid ground cover can reduce evaporation losses by shading the soil (López-Castañeda and Richards, 1994), increase total photosynthesis by extending the duration of light capture (Parry et al., 2011) and enhance weed competitiveness of the crop (Bertholdsson, 2005). Our results showed a significant association of early vigor with water use only in the dry year 2012, suggesting secondary associations of this trait with phenology and root length density (Figure 2). With respect to inter-trait relations, however, results should be treated with caution if the data are concentrated at the two ends of the regression line. The associations might be a consequence of constitutive differences between underutilized wheat species and modern varieties rather than expressing causal inter-trait relations.

#### Water use efficiency traits

Crop growth depends on acquiring CO_2_ through open stomata, which in turn results in water loss through transpiration. Although upscaling from stomata gas exchange (intrinsic WUE) to whole plant WUE is complex (Hsiao et al., 2007), suitability of stomatal conductance as selection criterion has been demonstrated under both drought stress and well watered conditions (Rebetzke et al., 2013b). In both years the early maturing durum “Matt” was among the genotypes with highest stomatal conductance, suggesting an association between earliness and/or crop growth rate with stomatal conductance. Araus et al. (2002) pointed to higher stomatal opening as a consequence of crop earliness and lower leaf area index (LAI). Also in our study stomatal conductance was significantly and negatively correlated with LAI in the dry year 2012 (*r* = −0.75, *p* < 0.05; data not shown). Contrary, late maturing *T. monococcum* and *T. timopheevii* had the lowest values of stomatal conductance. An influence of ploidy level on stomata characteristics with diploid species, having the smallest stomata, was demonstrated by Khazaei et al. (2010). Low stomata conductance of einkorn and Zanduri wheat suggested a conservative gas exchange strategy. Their comparatively high water use is, therefore, explained rather by prolonged duration of transpiration than a high rate of water extraction due to conductive stomata.

Stomatal conductance and photosynthetic capacity, traits underlying intrinsic WUE (Condon et al., 2002), seem to be strongly related to constitutive differences resulting from different breeding intensities. Similar to other studies, we found a significant association between stomata conductance and photosynthetic capacity (2011: *r* = 0.73, *p* < 0.01; 2012: *r* = 0.65, *p* = 0.058). This indicates a tight functional link between stomata opening ensuring high CO_2_ inflow and photosynthetic capacity providing efficient fixation of available carbon in modern high yielding varieties. It also confirms the challenge of improving intrinsic WUE by lower stomata conductance without compromising crop productivity (Blum, 2005; Lawson et al., 2012). Fischer et al. (1998) demonstrated the association of leaf photosynthetic rate and stomatal conductance with yield progress in CIMMYT wheat genotypes. Also Reynolds et al. (1994) reported a significant association between photosynthetic rate and stomatal conductance with grain yield. Combining stomata conductance and leaf chlorophyll content measurements could allow the identification of germplasm combining improved WUE and productivity under both well watered or water limited conditions (Rebetzke et al., 2013b).

#### Harvest index traits

Genetic variation in harvest index within our germplasm was largely determined by distinct differences in yield components and phenology (Figure 2). Unlike modern cultivars, underutilized wheat species were more dependent on alteration of assimilate allocation between root and shoot in response to drought (Nakhforoosh et al., 2014). The observed association between harvest index and root to shoot in the second year most probably results from an intrinsic low harvest index of the underutilized wheat species resulting from their high allocation to roots under limited water availability.

**Figure 5:**
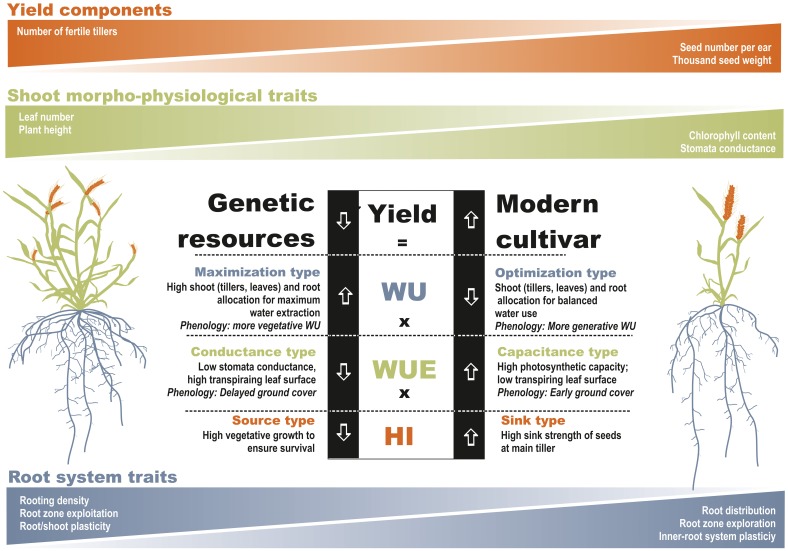
**Distinctive behavior of wheat genetic resources and/or underutilized wheat species vs. modern varieties within Passioura's yield-water framework**. (Root trait differentiation is based on Nakhforoosh et al., 2014).

Dissecting the investigated germplasm according to Passioura's yield-water framework resulted in two contrasting patterns (Figure 5). Underutilized wheat species can be considered as maximization types in terms of water use. Their phenology and morphology allows an intensive water extraction as a basis for pronounced vegetative growth. This seems to be a safety strategy based on a high number of tillers. Although the vegetative apparatus may suffer a high reduction of tillers in case of later water limitation, still the crop will avoid complete failure. Contrary, modern varieties are optimized with respect to effective water use which is well balanced between vegetative and generative demand. This strategy is most appropriate to sustainably supply less but still highly demanding generative sinks. In case of high water stress, this strategy may be risky and result in total crop failure if not sufficient water for their main yield components is available.

In terms of WUE underutilized wheat species can be defined as conductance types and modern varieties as capacitance types. The high conductance, however, does not refer to the stomata scale but to the whole plant scale. The intense vegetative apparatus with high leaf area results in a high transpiring surface. This goes along with a low stomatal conductance and low photosynthetic capacity, both limiting assimilation potential. In modern varieties high stomatal conductance is linked to high photosynthetic capacity which ensures an efficient supply of assimilates. Water losses are controlled by an optimized total leaf area, ensuring sufficient light interception while avoiding unnecessarily high transpiring surface.

Differences in harvest index between old and modern varieties are well documented. We characterized the distinctive pattern as source types for underutilized wheat with an extensive vegetative apparatus and as sink type for modern varieties where available resources are efficiently allocated to a strong generative sink.

## Conclusion

Our study demonstrated that underutilized wheat species with low or no breeding intensity show serious limitations as source of novel traits of potential interest for wheat improvement. Their main strength is an efficient root water extraction linked to high assimilate translocation to roots, high tillering capacity, and long vegetative growth. In modern high yielding cultivars physiological traits such as stomata conductance combined with leaf chlorophyll concentration are responsible for their superior performance in well watered and stress conditions. The high yield stability of *T. turanicum* provides evidence that, despite limited yield potential, also some underutilized genetic material can be a source of interesting adaptive processes for future trait based breeding with respect to drought tolerance. Passioura's yield-water framework provides an appropriate conceptual model to guide such trait based analysis of breeding material. Our overall results suggest that crop improvement in water limited environments will likely profit more from making use of unexploited secondary traits in modern varieties than relying on wide crosses. Khorasan wheat, however, demonstrated that landraces or landrace selections of wheat subspecies of the same ploidy level may reveal promising drought stress response strategies that are currently not present in modern varieties.

### Conflict of interest statement

The authors declare that the research was conducted in the absence of any commercial or financial relationships that could be construed as a potential conflict of interest.
