# Supplementary material for: Dissection of drought response of modern and underutilized wheat varieties according to Passioura's yield-water framework
Source: Front Plant Sci. 2015 Jul 23;6:570. doi: 10.3389/fpls.2015.00570 (PMC4511830; doi:10.3389/fpls.2015.00570)
Supplement: Supplementary file 1 [file Table1.DOCX]

***Supplementary Material***

**Dissection of drought response of modern and underutilized wheat varieties according to Passioura’s yield-water framework**

**Alireza NAKHFOROOSH^1*^, Heinrich GRAUSGRUBER^1^, Hans-Peter KAUL^1^, Gernot BODNER^1^**

^1^Department of Crop Sciences, University of Natural Resources and Life Sciences, Vienna, Austria.

***Correspondence:** Alireza Nakhforoosh, University of Natural Resources and Life Sciences, Vienna

Department of Crop Sciences, Konrad-Lorenz-Str. 24, 3430 Tulln, Austria.

[alireza.nakhforoosh@boku.ac.at](mailto:alireza.nakhforoosh@boku.ac.at)

1. **Supplementary Figures and Tables**

## Supplementary Figures

**
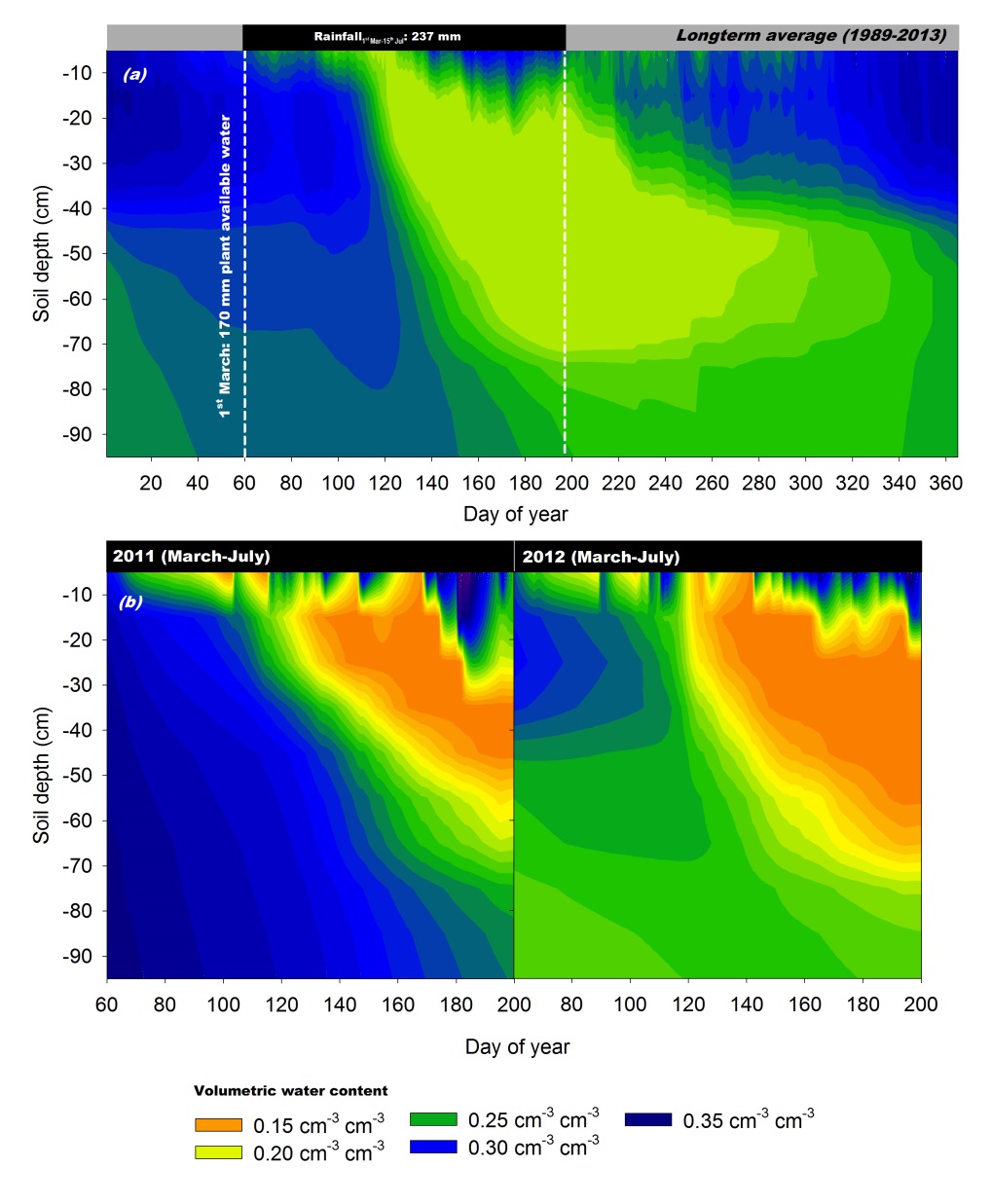
**

**Supplementary Figure 1.** Simulated spatio-temporal dynamics of soil profile water content using the simulation model HYDRUS 1D for **(a)** long term average conditions (1989 to 2013) and **(b)** for the two growing seasons of 2011 and 2012 at the experimental site. Dash lines in **(a)** specify the range of the growing season for spring cereals.

## Supplementary Tables

**Supplementary Table 1.** Summary of the selected linear mixed models of the analyses of variance of the core set genotypes across the two experimental years. Significant (*P*≤0.05) fixed effects are printed in bold capital.

| **Trait** | **Fixed effects^1^** | **Random effects^1^** |
| --- | --- | --- |
| Water use | **G** | B(Y) + Y |
| Time to full flowering (thermal time based) | **G** | B(Y) + Y + G×Y |
| Time to full flowering (calendar time based) | **G** | Y + G×Y |
| Early vigor | g | Y + G×Y |
| Leaf chlorophyll content (SPAD) | **G** | B(Y) + G×Y |
| Stomatal conductance | g | Y+ G×Y |
| Water use efficiency for biomass | **G** | Y |
| Seed yield | **G** | Y+ G×Y |
| Shoot biomass | **G** | Y |
| Harvest index | **G** | Y + G×Y |
| Number of fertile tillers | g | B(Y) + G×Y |
| Seed number per ear | **G** | Y + G×Y |
| Thousand kernel weight | **G** | Y + G×Y |
| Plant height | g | B(Y) + G×Y |

^1^ B, block; G, genotype; Y, year

**Supplementary Table 2.** Mean values of Passioura’s yield determining components for the two main distinctive genetic groups

| **Year** | **Genotype group** | **Water use (mm)** | **Water use efficiency (g m^-2^ mm^-1^)** | **Harvest**  **index** | **Yield (g m^-2^)** |
| --- | --- | --- | --- | --- | --- |
| **2011** | Modern cultivars^1^ | 233.6 | 4.21 | 0.45 | 445.5 |
|  | Genetic resources^2^ | 275.2 | 3.43 | 0.26 | 248.1 |
| **2012** | Modern cultivars | 151.1 | 3.01 | 0.46 | 208.3 |
|  | Genetic resources | 168.6 | 1.96 | 0.13 | 41.7 |
| **RSR^3^** | Modern cultivars | 35.3 | 28.5 | -0.20 | 53.2 |
|  | Genetic resources | 38.7 | 42.9 | 50.0 | 83.2 |

^1^ Floradur, Matt – *Triticum durum*

^2^ W9 – *T. timopheevii*; PI428154, PI428165 – *T. monococcum*

^3^ RSR, relative stress response (formula see Materials & methods)
